# Adaptations for remote research work: a modified web-push strategy compared to a mail-only strategy for administering a survey of healthcare experiences

**DOI:** 10.1186/s12874-023-02066-5

**Published:** 2023-10-19

**Authors:** Varsha G. Vimalananda, Jolie B. Wormwood, Kailyn E. Sitter, B. Graeme Fincke, Shirley Qian, Maya N. Tait, Mark Meterko

**Affiliations:** 1 Center for Healthcare Organization and Implementation Research (CHOIR), VA Bedford Healthcare System, 200 Springs Road, Building 70, Bedford, MA 01730 USA; 2 Section of Endocrinology, Diabetes and Metabolism, Boston University School of Medicine, Boston, MA USA; 3 Department of Psychology, University of New Hampshire, Durham, NH USA; 4 Department of Health Law, Policy and Management, Boston University School of Public Health, Boston, MA USA; 5 International School of Boston, Cambridge, MA USA; 6 Analytics and Performance Integration, Office of Quality and Patient Safety, Department of Veterans Affairs, Bedford, MA USA

**Keywords:** Survey research, Patient surveys, Specialty care, Veterans

## Abstract

**Background:**

The COVID-19 pandemic required that our research team change our mail-only (MO) strategy for a research survey to a strategy more manageable by staff working remotely. We used a modified web-push approach (MWP), in which patients were mailed a request to respond online and invited to call if they preferred the questionnaire by mail or phone. We also changed from a pre-completion gift to a post-completion gift card incentive. Our objective is to compare response patterns between modes for a survey that used an MO strategy pre-pandemic followed by an MWP strategy peri-pandemic for data collection.

**Methods:**

Observational study using data from a national multi-scale survey about patients’ experience of specialty care coordination administered via MO in 2019 and MWP from 2020 to 2021 to Veterans receiving primary care and specialty care within the Veterans Health Administration (VA). We compared response rates, respondent characteristics and responses about care coordination between MO and MWP, applying propensity weights to account for differences in the underlying samples.

**Results:**

The response rate was lower for MWP vs. MO (13.4% vs. 36.6%), OR = 0.27, 95% CI = 0.25–0.30, P < .001). Respondent characteristics were similar across MO and MWP. Coordination scale scores tended to be slightly higher for MWP, but the effect sizes for these differences between modes were small for 9 out of 10 scales.

**Conclusions:**

While the logistics of MWP survey data collection are well-suited to the remote research work environment, response rates were lower than those for the MO method. Future studies should examine addition of multi-mode contacts and/or pre-completion incentives to increase response rates for MWP.

**Supplementary Information:**

The online version contains supplementary material available at 10.1186/s12874-023-02066-5.

## Introduction

The COVID-19 pandemic impacted research data collection in multiple ways, including for patient surveys. When the pandemic struck, our research team had recently completed pilot administration of a mail-only (MO) national survey of patients’ experience of specialty care coordination. We had used Dillman’s multi-contact approach [1] and included a small gift with the request. As the pandemic persisted, we were compelled to continue the planned large-scale data collection but needed to account for research staff working now almost entirely remotely. It was not feasible for remote staff to carry hundreds of paper questionnaires home to assemble mailings, track returned mail and completed paper questionnaires in time to avoid unnecessary repeat contacts, or scan completed paper questionnaires using on-site equipment and software. These conditions necessitated modification of our approach to rely heavily on online surveys. However, due to research regulations, we could not use patient email addresses to initiate contact.

We first considered a web-push strategy [1]. With web-push, individuals are invited by mail to respond to an online survey. Later in the process, outbound mail and phone calls offer those modes for completion. Dillman et al. conducted a series of seminal experiments between 2007 and 2012 to understand respondents with web-push and what strategies were most effective for that mixed mode [2–6]. They found that the combined mail and web respondents in web-push are demographically similar to mail-only respondents, with the later paper questionnaire bringing in respondents with characteristics not well represented among web respondents [7]. Typically, the incentive is sent with the initial web-push request to provide motivation for transitioning from the letter to typing a URL. An incentive enclosed with the web-push request was shown to increase the response rate by 18% points in one of these studies [5]. However, to better meet the conditions of remote work, we made two modifications to these practices. First, to reduce the burden of mailing, tracking, and scanning paper responses, we invited patients to call if they wanted to complete the questionnaire via mail or phone, rather than presumptively sending out paper questionnaires. We also switched the incentive from a bag/notepad/pen included with the request to a post-completion gift card, to reduce the volume of materials managed by staff. While these methods are optimized to accommodate research staff working at different sites, often away from the office, such an approach has not been evaluated in other research. Therefore data on the promise and shortcomings of these changes for future studies is unclear.

Below, we describe our results using a modified web-push (MWP) strategy adapted for remote work. We compare MO and MWP using a large-scale national survey of Veterans’ experience of care coordination that utilized both modes of administration. We address three questions:

1. Is there a difference in the response rate for a mail-only (MO) compared to a modified web-push (MWP) strategy?
2. Are there differences in respondent demographic characteristics by survey mode?
3. Are there differences by survey mode in answers concerning healthcare experience?

## Methods

The VA Bedford Healthcare System Institutional Review Board approved this study and its waiver of informed consent. All methods were carried out in accordance with relevant guidelines and regulations.

### Sample

As part of an observational research study on experiences of coordination between primary and specialty care, we administered a survey [8] to patients receiving care through the Veterans Health Administration (VA), a large integrated health system. The questionnaire is the patient version of measures previously developed for PCPs [9] and specialists [10, 11]. We used the VA Corporate Data Warehouse (CDW) to identify patients aged ≥ 18 who, in the six months prior, had [1] seen a clinician in one of eight medical subspecialties included in the study, and [2] seen their PCP before and after the index specialty care visit.

### Questionnaire content

We used the previously-developed Coordination of Specialty Care – Patient Survey, which includes 39 items across 10 multi-item scales that measure the patient experience of specialty care coordination. Constructs measured by the questionnaire are Patient-Centered Care Coordination, Specialist Communication, Access to Specialist Care, Specialist Knowledge of Patient History, Referral Shared Decision Making, Tests & Medications: Patient Information, Tests & Medications: Get Results, Overall Trust & Specialty Care Engagement of PCP, Coordination of Care – Specialist & PCP, and Team Planning for Patient Self-Care. Scale scores range from 1 to 5 (from disagree to agree). Data on internal consistency reliabilities (Cronbach’s alpha) and inter-scale correlations (zero order Pearson’s correlations) are available in Additional File 1. We included a single-item measure of overall satisfaction with VA health care (1–6 scale from very dissatisfied to very satisfied), self-reported physical and mental health status (1–5 scales from poor to excellent), and demographic items.

### Data collection

We used an MO administration for the pilot study. By the time we completed analysis of pilot data and were ready to complete the study, the COVID-19 pandemic was well underway. We pivoted to the MWP strategy for the large launch. Both administrations used the same invitation letter, study fact sheet, and opt-out postcards.

### Mail only (MO) – pilot administration

From August-November 2019 we sent up to 3 mailings to a sample of 527 patients. The first mailing included a notification letter and postage-paid opt-out card. The second mailing included a cover letter, a study fact sheet, the questionnaire with a pre-paid return envelope, an opt-out card, and the incentive of a VA-branded notepad, pen, and tote bag. The third mailing included a reminder/thank you letter, study fact sheet, and the questionnaire with a pre-paid return envelope.

### Modified web-push (MWP) – large launch

From September 2020-May 2021 we contacted 5,288 Veterans by mail up to 3 times. Each mailing included an invitation letter, study fact sheet, and postage-paid opt-out card (first two mailings only). The letter contained the questionnaire URL (shortened for accessibility using tinyurl.com) and a QR code. Reminder letters provided the option of calling to complete the questionnaire by phone or request it mailed. Participants were sent a $10 gift card to a national pharmacy chain upon our receipt of their completed survey. Online responses were collected by Qualtrics; study staff input paper and phone responses into the Qualtrics database including a flag variable that distinguished these respondents.

### Statistical analysis

We compared the MO and MWP samples (respondents and non-respondents) on demographic characteristics from CDW: sex, age, rural/urban status, and the VA Care Assessment Needs (CAN) score [12], which identifies patients at high risk for hospitalization and mortality. We also compared respondents only on five characteristics from the survey: education, self-reported physical health, self-reported mental health, having help completing the survey, and overall satisfaction with VA care. We used independent samples t-tests, Wilcoxon two-sample tests, and chi-square tests as appropriate.

We compared raw response rates and rates after controlling for demographic differences between the two samples. We calculated response rates in a manner aligned with AAPOR recommendations [13]. The numerator was calculated as (Complete responses + Partial responses). The denominator was calculated as (Complete responses + Partial responses) + (Refusal and Breakoff + Non-contact + Other) + (Unknown Household/Unknown Other). For these response rate comparisons only, we controlled for demographic differences in the samples by calculating and applying propensity score weights using the five CDW-based demographic characteristics. Propensity scores were calculated by first running a binary logistic regression predicting survey mode from the five CDW variables simultaneously. The predicted value for each participant calculated from this model was taken as the propensity score. Weights of 1/propensity score were applied to the MWP sample and weights of 1/(1-propensity score) were applied to the MO sample. Applying the propensity scores as weights in this manner equates the samples on these covariates and is a preferred method for controlling for extraneous variables when sample size is small and/or the number of control variables is large [14]. Supplemental analyses compared the demographic variables used in calculating propensity scores across samples with propensity score weights applied using independent samples t-tests or chi-square tests as appropriate. These supplemental analyses (Additional File 2) confirmed that weighting successfully controlled for demographic differences between samples. For MWP respondents we reported the frequency of final responses by mode.

Although controlling for demographic differences was important for a fair comparison of response rates, we also wanted to evaluate whether the two survey modes produced respondents with different demographics, as this provides information about the potential for non-response bias. We compared demographics of respondents to non-respondents within each mode separately using independent samples t-tests, Wilcoxon two-sample tests, and chi-square tests, and then we conducted separate binary logistic regression analyses, one for each demographic variable, to examine whether there were significant interactions between each demographic characteristic and mode in predicting the likelihood of responding. These analyses tell us whether any associations between demographic characteristics and the likelihood of responding differ across modes (e.g., whether the relationship between responding and age differed for MO vs. MWP).

Finally, we examined whether survey mode led to a different respondent profile in terms of experience of care coordination. We compared coordination scale scores between MO and MWP respondents with independent samples t-tests using both raw scale scores and propensity score-weighted scale scores. For these analyses among respondents only, a second, separate set of propensity scores were calculated and applied. Here, propensity scores were calculated to balance the two respondent sub-samples using all ten Table 1 variables. Supplemental analyses (Additional File 2) confirmed that these new propensity weights successfully controlled for differences on all 10 variables from Table 1 across respondent sub-samples. In addition, we calculate and report effect size estimates for these comparisons (Cohen’s *d*) and interpret the values as small, moderate, or large based on established standards [15].

## Results

### Patient characteristics

The top section of Table 1 provides descriptive statistics for the two samples (respondents and non-respondents). The MWP sample was younger, healthier, and included a higher proportion of female and lower proportion of married people (all *p* < .001). The bottom section of Table 1 provides descriptive statistics for self-reported characteristics among respondents. MWP respondents were more likely to have had at least a college education and were less satisfied overall with their VA care (both *p* < .01).

**Table 1:** Patient characteristics among the survey administration samples and respondents by survey mode (N = 5,815)

	Source: Administrative Data – Respondents and Non-Respondents (N = 5,815)		
	Mail-only sample (N = 527)	MWP sample* (N = 5288)	*p-value*
Female *[N, (%)]*	42 (8.0%)	820 (15.5%)	< 0.001^a^
Age *[Mean (SD)]*	66.9 (12.6)	64.3 (13.6)	< 0.001^b^
Married *[N, (%)]*	309 (58.6%)	2324 (44.0%)	< 0.001^a^
Rural residence *[N, (%)]*	373 (71.2%)	3841 (72.6%)	0.135^a^
CAN score *[Mean (SD)]*^†^	64.8 (28.6)	59.0 (30.8)	< 0.001^b^
	**Source: Self-Report –** **Respondents Only (N = 909)**		
	**Mail-only sample respondents (N = 197)**	MWP sample* respondents (N = 712)	*p-value*
At least some college *[N, (%)]*	118 (59.9%)	513 (72.1%)	< .0001^a^
Self-reported physical health^‡^*[Mean (SD)]*	2.6 (0.97)	2.6 (1.00)	0.90^c^
Self-reported mental health^‡^*[Mean (SD)]*	3.0 (1.19)	3.1 (1.22)	0.49^c^
Received help to complete survey *[N, (%)]*	25 (12.7%)	101 (14.2%)	0.60^a^
Overall satisfaction with VA care *[Mean (SD)]*^§^	5.1 (1.28)	4.8 (1.47)	0.01^c^

*MWP: modified web-push

^†^CAN score can range from 0–99

^‡^self-reported physical and mental health are on a scale from 1–5

^§^overall satisfaction with VA care is on a scale of 1–6

^a^Chi-square test

^b^Independent samples t-test

^c^Wilcoxon two-sample Test

### Response rates

The MWP response rate (13.5%) was about half that of the MO response rate (37.4%). Among MWP respondents, 83% responded online, 12% by mail, and 5% by telephone. After propensity weighting, the MWP response rate (13.4%) remained significantly lower than the MO response rate (36.6%) (*OR* = 0.27, *95%CI* = 0.25–0.30, *p* < .001).

### Associations between demographic characteristics and responding

We conducted moderator (i.e., interaction) analyses to examine whether any associations between demographic variables and responding (vs. not) differed across the two samples. We found no significant moderation of associations by survey mode (see Table 2, p-values in rightmost column). For example, respondents were more likely to be married than non-respondents, respectively, in both the MO sample (63.5% v. 55.2%) and MWP sample (50.7% v. 43.7%), and the moderator analyses revealed these differences did not differ significantly between samples, *p* = .77. Similarly, respondents had lower CAN scores on average than did non-respondents in both the MO (*M* = 54.8 v. *M* = 59.4) and the MWP samples (*M* = 63.1 v. *M* = 66.2), and these differences did not differ significantly between samples, *p* = .63.

**Table 2:** Comparisons of the demographic characteristics of respondents and non-respondents by survey mode

Survey Mode	Mail-Only Sample (N = 527)			MWP^†^ Sample (N = 5288)			*Difference in Association Across Survey Mode* *(p-value)*
	Non-respondents (N = 330)	Respondents (N = 197)	*p-value*	Non-respondents (N = 4576)	Respondents (N = 712)	*p-value*	
Female [N(%)]	27 (8.2%)	15 (7.6%)	0.82^a^	691 (15.1%)	129 (18.1%)	0.04^a^	**.40** ^**c**^
Age [M (SD)]	66.3 (13.6)	67.8 (10.6)	0.16^b^	864.2 (13.9)	64.9 (12.7)	0.22^b^	**.55** ^**c**^
Married [N(%)]	184 (55.3)	125 (63.5%)	0.08^a^	1967 (43.3%)	357 (50.7%)	0.000^a^	**.92** ^**c**^
Rural [N(%)]	96 (29.4%)	55 (27.9%)	0.72^a^	1149 (25.6%)	187 (26.9%)	0.48^a^	**.54** ^**c**^
CAN score [M (SD)]	65.8 (28.9)	63.1 (28.2)	0.29^b^	59.7 (31.0)	54.8 (29.2)	.0001^b^	**.48** ^**c**^

^†^MWP: modified web-push

^a^Chi-square test

^b^Independent samples t-test

^c^p-value associated with interaction term in binary logistic regression

### Respondents’ experiences of care coordination by survey mode

In both the raw comparisons and propensity-weighted comparisons, mean scores for two of 10 scales were higher in the MWP group (Table 3). Propensity-weighted comparisons showed significantly higher scores for two additional scales.

To better understand the meaning of these findings, we examined effect sizes (Cohen’s *d* values) for each difference (Table 3, rightmost column). Out of the 10 weighted scale score comparisons only one demonstrated a moderate effect size (difference in mean score 0.61 on 1–5 scale; Cohen’s *d* = 0.37). For the other 9 scales, differences in mean scores ranged from 0.08 to 0.24 and Cohen’s *d* for these scales ranged from 0.06 to 0.19, all representing small effect sizes.

**Table 3:** Comparison of scale scores (experiences of care coordination) by survey mode*

	Raw Data			Weighted by Propensity Score			
	**Mail-Only Respondents** **(N = 197)**	**MWP Respondents** ^†^ **(N = 712)**	***p-value*** ^***a***^	**Mail-Only Respondents** **(N = 197)**	**MWP Respondents** ^†^ **(N = 712)**	***p-*** ***value*** ^***a***^	**Cohen’s** ***d***
Patient-Centered Coordination of Care	3.86	3.91	0.39	3.81	3.94	0.07	**0.11**
Specialist Communication	4.09	4.21	0.83	4.00	4.23	0.04	**0.13**
Access to Specialist Care	3.78	3.88	0.20	3.75	3.91	0.05	**0.13**
Coordination of Care: Specialist with Patient	4.00	4.00	0.89	3.95	4.03	0.31	**0.06**
Referral Shared Decision Making	4.03	4.20	0.03	4.04	4.21	0.03	**0.15**
Tests & Medications: Patient Education	4.00	4.12	0.14	3.91	4.15	0.009	**0.19**
Tests & Medications: Get Results	4.00	4.07	0.40	3.93	4.12	0.06	**0.14**
Overall Trust & Specialty Care Engagement of PCP	3.86	4.35	< 0.0001	3.78	4.38	< 0.0001	**0.37**
Coordination of Care – Specialist and PCP	3.50	3.56	0.49	3.42	3.58	0.06	**0.12**
Team Planning for Patient Self-Care	3.44	3.44	0.96	3.34	3.47	0.18	**0.09**

*theoretical range 1–5 for all scale

^†^MWP: modified web-push

^a^All p-values from independent samples t-tests

## Discussion

To accommodate newly remote staff during the COVID-19 pandemic, we changed our survey data collection strategy from MO with a pre-completion bag/notepad/pen incentive to a MWP strategy with a post-completion gift card incentive. We used data from a patient survey about VA specialty care coordination that used both strategies to understand how MWP is associated with response rates, respondent characteristics, and responses.

The MWP response rate was more than 20% lower than that for MO, even after weighting. This difference is larger than those in older studies reporting lower web-push response [16] and contrasts with newer studies reporting similar or higher response rates for web-push compared to mail-only [17–19]. There are several potential reasons for the lower-than-anticipated MWP response rate. There is evidence of lower response rates peri-pandemic, which some authors attribute to ‘survey fatigue’ from a rise in survey-based research methods to enable remote data collection during the pandemic [20]. Also, while we were able to sustain certain practices for MWP that are associated with higher response rates, including prenotification [21, 22], mixed modes [23, 24], offering mixed modes sequentially rather than concurrently [25, 26], using multiple contacts [27, 28], and use of incentives [29, 30], we made other modifications to accommodate remote work that may have had a negative impact on response rates.

In general, web-based surveys are associated with lower response rates than mailed surveys [31–33] – by an average of 11% points, according to one meta-analyses of 114 experimental studies [34]. While there are conflicting data about the most effective mode for the invitation to a web survey, the same meta-analysis suggested solicitation to web survey by a mode other than email, as was the case for our project, was associated with a response rate decrease of 8% points [34]. This may be in part due to the extra effort required to read and type a URL into a web browser or scan a QR code rather than simply click an email-embedded link. The MWP gift card incentive was conditional on survey completion, which may have been less effective because it invoked a payment that may not have felt adequate, as compared to the MO gift provided in advance, which may have invoked a social exchange and created a desire to reciprocate a feeling of goodwill [1, 35]. A final, major difference between MWP and typical web-push is that we did not automatically send paper surveys or make phone calls to web survey non-respondents. Following a web survey with mail can result in a response rate close to that of mail alone − 51% mail-only vs. 49% web-push in one study [36] and 43% mail-only vs. 41% web-push in another [31].

Differences in underlying samples for the two modes likely reflect a shift in the demographics of patients who continued VA care during the early pandemic. Propensity weighting successfully controlled for differences in the underlying samples and resulted in no statistically significant differences between the two modes in respondent characteristics. This is an indirect way to assess non-response bias, but suggests that different non-response bias was not introduced by the MWP mode, and that we have identified variables that can be used for successful adjustment for patient characteristics in propensity weighting. Successful adjustment allows for combining data from administrations using MO and MWP into a single dataset for analyses.

While we found some differences between mode in coordination scores, the effect sizes for the propensity-weighted differences were all small, except for one scale. Whether these score differences are related to the different timing of the two modes or the modes of administration themselves is a question that can be addressed by simultaneous use of the MO and MWP in future work.

Given that remote research is here to stay, the question is how we might integrate additional best practices to boost response rates for MWP while minimizing the volume of materials handled in typical web-push. Simply administering the survey as MWP outside of the acute phase of a pandemic may result in improved response rates. Unfortunately, as researchers we are unlikely to obtain permission to use patient email addresses for survey invitations. However, we could include a pre-completion gift card incentive rather than a pre-completion gift or post-completion incentive. We could also contact non-respondents by phone and offer the option for survey completion by phone or mail. Automatically mailed questionnaires could be used, but minimized by reserving them for non-respondents to all other contacts.

Our study has limitations. Our change in mode partway through was necessitated by the need to quickly adapt to the impact of the COVID-19 pandemic on our research. Our results should thus be interpreted with an understanding of the limitations of an observational rather than an experimental design. For example, while we did account for differences in the underlying sample at the two timepoints using propensity scores, there may have been other differences related to pandemic-related experience that we were not able to control for and that impacted response rates, respondent characteristics, and experience of care coordination. Because we made a few changes at once as we pivoted our approach, it is not possible to tease out the independent effect of any one change. Despite these limitations, our results add value because they describe one approach to survey administration for a remote work environment, which is increasingly common. The results indicate no difference in non-response bias, supporting a rationale for testing improvements to MWP outside of a pandemic setting and drawing from existing literature, to improve response rates with a remote work-aligned approach to large-scale surveys.

## Conclusion

MWP, with its lower burden of physical materials, is well-suited to a remote work environment and did not result in respondent characteristics different from MO. However, we observed a much lower response rate than expected based on prior literature and it is unclear whether responses themselves (i.e., ratings of experience) may differ by mode. These findings underscore the value of piloting any new survey method, because response rates may be unexpected as observed in this study. Future work should examine whether higher response rates with MWP could be achieved when pandemic stress is less acute than it was during this study, by providing a pre-completion gift card, and/or by adding modes of contact. Simultaneous testing of different strategies may eliminate the need for propensity weighting and alleviate concerns about a secular effect on responses.

### Electronic supplementary material

Below is the link to the electronic supplementary material.

Supplementary Material 1

Supplementary Material 2

## Data Availability

The datasets used and/or analyzed during the current study are available from the corresponding author on reasonable request.

## Abbreviations

MO: Mail-only
MWP: Modified web-push
VA: Veterans Health Administration
CDW: Corporate Data Warehouse
CAN: VA Care Assessment Needs

## Declarations

Acknowledgments:
