# Supplementary material for: Adaptations for remote research work: a modified web-push strategy compared to a mail-only strategy for administering a survey of healthcare experiences
Source: BMC Med Res Methodol. 2023 Oct 19;23:244. doi: 10.1186/s12874-023-02066-5 (PMC10588167; doi:10.1186/s12874-023-02066-5)
Supplement: Supplementary file 1 — Supplementary Material 1 [file 12874_2023_2066_MOESM1_ESM.docx]

**Additional File 1.** **Correlations Among Scales of the Coordination of Specialty Care Survey – Patient Version (CSC – Patient)***

|  | **Patient-Centered Coordination of Care** | **Specialist Communication** | **Access to Specialist Care** | **Specialist Knowledge of Patient History** | **Referral Shared Decision Making** | **Tests & Medications: Patient Education** | **Tests & Medications: Get Results** | **PCP: Overall Trust & Specialty Care Engagement of PCP** | **Coordination of Care – Specialist and PCP** | **Team Planning for Patient Self-Care** |
| --- | --- | --- | --- | --- | --- | --- | --- | --- | --- | --- |
| Patient-Centered Care Coordination | (0.96) |  |  |  |  |  |  |  |  |  |
| Specialist Communication | 0.87 | (0.94) |  |  |  |  |  |  |  |  |
| Access to Specialist Care | 0.64 | 0.61 | (0.77) |  |  |  |  |  |  |  |
| Coordination of Care: Specialist with Patient | 0.72 | 0.71 | 0.54 | (0.84) |  |  |  |  |  |  |
| Referral Shared Decision Making | 0.57 | 0.56 | 0.62 | 0.48 | (0.69) |  |  |  |  |  |
| Tests & Medications: Patient Education | 0.80 | 0.73 | 0.56 | 0.62 | 0.48 | (0.83) |  |  |  |  |
| Tests & Medications: Get Results | 0.79 | 0.71 | 0.57 | 0.62 | 0.50 | 0.73 | (0.87) |  |  |  |
| PCP: Overall Trust & Specialty Care Engagement of PCP | 0.57 | 0.54 | 0.49 | 0.41 | 0.43 | 0.55 | 0.60 | (0.90) |  |  |
| Coordination of Care – Specialist and PCP | 0.48 | 0.43 | 0.36 | 0.44 | 0.30 | 0.36 | 0.44 | 0.54 | (0.85) |  |
| Team Planning for Patient Self-Care | 0.63 | 0.53 | 0.49 | 0.50 | 0.40 | 0.48 | 0.54 | 0.68 | 0.54 | (0.86) |
|  |  |  |  |  |  |  |  |  |  |  |
| Single item about Overall Coordination | 0.62 | 0.55 | 0.49 | 0.49 | 0.36 | 0.46 | 0.55 | 0.65 | 0.61 | 0.74 |

*Internal consistency reliability in parentheses in the diagonal
