# Supplementary material for: Adaptations for remote research work: a modified web-push strategy compared to a mail-only strategy for administering a survey of healthcare experiences
Source: BMC Med Res Methodol. 2023 Oct 19;23:244. doi: 10.1186/s12874-023-02066-5 (PMC10588167; doi:10.1186/s12874-023-02066-5)
Supplement: Supplementary file 2 — Supplementary Material 2 [file 12874_2023_2066_MOESM2_ESM.docx]

**Additional File 2. Effectiveness of propensity score weighting**

**Table A. Patient characteristics among the fully mailed and push-to-web survey administration samples (*N*=5815). Comparisons on raw and propensity score weighted data.**

|  | **Raw Data** | | | **Weighted by Propensity Score** | | |
| --- | --- | --- | --- | --- | --- | --- |
|  | **Fully mailed sample**  **(N=527)** | **Push-to-web sample (N=5288)** | ***p-value*** | **Fully mailed sample**  **(N=527)** | **Push-to-web sample (N=5288)** | ***p-value*** |
| Female *[N, (%)]^a^* | 42 (8.0%) | 820 (15.5%) | <0.001 | (13.2%) | (14.5%) | .048 |
| Age *[Mean (SD)]^b^* | 66.9 (12.6) | 64.3 (13.6) | <0.001 | 65.2 (43.5) | 64.8 (14.0) | .530 |
| Married *[N, (%)]^a^* | 309 (58.6%) | 2324 (44.0%) | <0.001 | (46.1%) | (45.2%) | .352 |
| Urban/rural residence *[N, (%)]^a^* | 373 (71.2%) | 3841 (72.6%) | 0.135 | (74.1%) | (73.9%) | .871 |
| CAN score *[Mean (SD)]^b^* | 64.8 (28.6) | 59.0 (30.8) | <0.001 | 60.5 (97.8) | 60.0 (32.0) | 0.708 |

^a^Chi-square test used

^b^Independent samples t-test used

**Table B. Patient characteristics among the fully mailed and push-to-web survey respondents only (*N*=909). Comparisons on raw and propensity score weighted data.**

|  | **Raw Data** | | | **Weighted by Propensity Score** | | |
| --- | --- | --- | --- | --- | --- | --- |
|  | **Fully mailed sample**  **(N=197)** | **Push-to-web sample (N=712)** | ***p-value*** | **Fully mailed sample**  **(N=197)** | **Push-to-web sample (N=712)** | ***p-value*** |
| Female *[N, (%)]^a^* | 15 (7.6%) | 129 (18.1%) | < .001 | (14.6%) | (16.1%) | .408 |
| Age *[Mean (SD)]^b^* | 67.8 (10.6) | 64.9 (12.7) | .004 | 65.6 (26.6) | 65.4 (13.7) | .875 |
| Married *[N, (%)]^a^* | 125 (63.5%) | 357 (50.7%) | .002 | (57.9%) | (54.5%) | .167 |
| Urban/rural residence *[N, (%)]^a^* | 55 (27.9%) | 187 (26.9%) | .778 | (28.9%) | (27.7%) | .581 |
| CAN score *[Mean (SD)]^b^* | 63.1 (28.23) | 54.8 (29.2) | < .001 | 56.2 (69.9) | 56.1 (32.2) | .947 |
| At least some college *[N, (%)]^a^* | 118 (59.9%) | 513 (72.1%) | <.0001 | (72.2%) | (74.6%) | .303 |
| Self-reported physical health^‡^ *[Mean (SD)]^c^* | 2.6 (0.97) | 2.6 (1.00) | 0.902 | 2.6 (2.1) | 2.6 (1.1) | .322 |
| Self-reported mental health^‡^ *[Mean (SD)]^c^* | 3.0 (1.19) | 3.1 (1.22) | 0.490 | 3.1 (2.5) | 3.1 (1.4) | .770 |
| Received help to complete survey *[N, (%)]^a^* | 25 (12.7%) | 101 (14.2%) | 0.605 | (17.9%) | (14.9%) | .228 |
| Overall satisfaction with VA care *[Mean (SD)]* ^§c^ | 5.1 (1.3) | 4.8 (1.5) | 0.012 | 4.9 (3.2) | 4.9 (1.6) | .200 |

^a^Chi-square test used

^b^Independent samples t-test used

^c^Wilcoxon Two-Sample Test
